# Biochemical characterization of adenosine deaminase (CD26; EC 3.5.4.4) activity in human lymphocyte-rich peripheral blood mononuclear cells

**DOI:** 10.1590/1414-431X2020e10850

**Published:** 2021-05-24

**Authors:** L.R. Costa, A.K.Y. de Souza, J.N. Scholl, F. Figueiró, A.M.O. Battastini, J.A. dos Santos Jaques, F.F. Zanoelo

**Affiliations:** 1 Laboratório de Bioquímica Geral e de Microrganismos, Instituto de Biociências, Universidade Federal de Mato Grosso do Sul, Campo Grande, Campo Grande, MS, Brasil; 2 Programa Multicêntrico de Pós-Graduação em Bioquímica e Biologia Molecular-SBBq, Instituto de Biociências, Universidade Federal de Mato Grosso do Sul, Campo Grande, MS, Brasil; 3 Departamento de Bioquímica, Instituto de Ciências Básicas e da Saúde, Universidade Federal do Rio Grande do Sul, Porto Alegre, RS, Brasil; 4 Programa de Pós-Graduação em Ciências Biológicas, Bioquímica, Instituto de Ciências Básicas e da Saúde, Universidade Federal do Rio Grande do Sul, Porto Alegre, RS, Brasil; 5 Programa de Pós-Graduação em Ciências Farmacêuticas, Faculdade de Farmácia, Alimentos e Nutrição, Universidade Federal de Mato Grosso do Sul, Campo Grande, MS, Brasil

**Keywords:** Adenosine deaminase, ADA, Lymphocyte-rich PBMCs, Enzymatic assays, Purinergic signaling

## Abstract

The conversion of adenosine to inosine is catalyzed by adenosine deaminase (ADA) (EC 3.5.4.4), which has two isoforms in humans (ADA1 and ADA2) and belongs to the zinc-dependent hydrolase family. ADA modulates lymphocyte function and differentiation, and regulates inflammatory and immune responses. This study investigated ADA activity in lymphocyte-rich peripheral blood mononuclear cells (PBMCs) in the absence of disease. The viability of lymphocyte-rich PBMCs isolated from humans and kept in 0.9% saline solution at 4-8°C was analyzed over 20 h. The incubation time and biochemical properties of the enzyme, such as its Michaelis-Menten constant (K_m_) and maximum velocity (V_max_), were characterized through the liberation of ammonia from the adenosine substrate. Additionally, the presence of ADA protein on the lymphocyte surface was determined by flow cytometry using an anti-CD26 monoclonal human antibody, and the PBMCs showed long-term viability after 20 h. The ADA enzymatic activity was linear from 15 to 120 min of incubation, from 2.5 to 12.5 µg of protein, and pH 6.0 to 7.4. The K_m_ and V_max_ values were 0.103±0.051 mM and 0.025±0.001 nmol NH_3_·mg^-1^·s^-1^, respectively. Zinc and erythro-9-(2-hydroxy-3-nonyl) adenine (EHNA) inhibited enzymatic activity, and substrate preference was given to adenosine over 2′-deoxyadenosine and guanosine. The present study provides the biochemical characterization of ADA in human lymphocyte-rich PBMCs, and indicates the appropriate conditions for enzyme activity quantification.

## Introduction

Adenosine deaminase (ADA; EC 3.5.4.4), represented in humans by two isoforms, ADA1 and ADA2, catalyzes the conversion of adenosine to inosine (and deoxyadenosine to deoxyinosine) (1–3). Both human ADA isoforms belong to the zinc-dependent hydrolase family and are strongly inhibited by deoxycoformycin and erythro-9-(2-hydroxy-3-nonyl) adenine (EHNA). Although ADA is present in all cell types, ADA levels differ widely among tissues. In humans, the highest ADA levels are found in lymphoid tissues (4), and ADA1 influences lymphocyte function. While certain inherited ADA mutations result in severe immunodeficiency, the physiological role of ADA2 is unknown. ADA2 is found in small amounts in the serum and can be produced by monocytes and macrophages (5).

In humans, ADA deficiency manifests primarily as severe lymphopenia and immunodeficiency, and, if untreated, can result in death by six months of age (4). Furthermore, it is associated with failure to thrive and opportunistic infections (4). Enzyme deficiency leads to adenosine (ADA substrate) accumulation, which may induce apoptosis and death in lymphocytes or developmental arrest (6).

The physiological effects of extracellular adenosine are mediated by its interaction with four G-protein-coupled receptors (A1, A2A, A2B, and A3) (7). These receptors mediate cellular signaling by protecting tissues and organs from damage through a variety of mechanisms that increase oxygen supply and demand, provoke anti-inflammatory effects, and stimulate angiogenesis. Studies related to adenosine receptors have shown applications in the treatment of pain, glaucoma, ischemic injuries, asthma, arthritis, cancer, inflammation, and other disorders (8).

Adenosine is considered a neuromodulatory and neuroprotective agent that also functions in other peripheral systems involved in the regulation of blood pressure, immune response, pain, and angiogenesis (3). Under conditions of energy deprivation, adenosine levels increase to influence energy homeostasis in various ways and regulate blood supply and cellular metabolism through maintaining membrane potentials (9).

The quantification of ADA activity has been previously performed in sheep lymphoid cells during antibody production after antigenic stimulation (10), peripheral blood cells of patients with hematological disorders, such as lymphoid leukemia and myeloma (11), serum from patients with different liver disorders (12), serum from patients with acquired immunodeficiency virus (HIV) infection (13), synovial fluid from patients with rheumatoid arthritis (14), serum from patients with tuberculous meningitis (15), serum from patients with chronic lymphoid leukemia (16), and other sources. ADA activity has also been characterized in leukemic cells (17), normal epidermal and carcinoma cells (18), mouse intestines (19), human and chicken livers (20), mollusk (*Biomphalaria glabrata*) hemolymph (21), and zebrafish brain (22). Although Giusti (23) and Guisti et al. (24) have characterized the enzymatic activity of ADA in human serum, no study has characterized ADA activity in isolated blood lymphocytes in detail, despite that many of the aforementioned studies quantified its activity in these specific cells.

Thus, the aim of this study was to standardize the enzymatic conditions (such as incubation time, protein content, temperature, pH, and ion requirement) and kinetic characteristics of human lymphocyte-rich peripheral blood mononuclear cells (PBMCs), in the absence of disease.

## Material and Methods

### Reagents

Adenosine, 2-deoxyadenosine, guanosine, EDTA, EHNA, Ficoll-Hypaque, Coomassie Blue G, and bovine serum albumin were obtained from Sigma-Aldrich (USA). All other chemicals used in this experiment were of analytical grade of the highest purity.

### Participants and ethical aspects

The Human Research Ethics Committee of the Federal University of Mato Grosso do Sul approved this research (protocol number: CAAE: 89595518.3.0000.0021). All participants provided written informed consent to participate in the study. The selected group included adults over 18 years of age from the Institute of Biosciences at the Federal University of Mato Grosso do Sul (InBio/UFMS), of both sexes, without reported chronic diseases (for example, high blood pressure and diabetes mellitus), autoimmune diseases, or acute infectious or inflammatory conditions at the time of blood collection. We also excluded smokers, drinkers, and pregnant women. Peripheral blood (approximately 10 mL per subject) was collected at the Section of Biochemistry (InBio/UFMS). The participants were randomly selected for the experiments, and four to six participants were required per experiment.

### PBMCs isolation

Human lymphocyte-rich PBMCs were isolated from peripheral blood collected with EDTA (3-4 mL) and separated by Ficoll-Hypaque density gradients, as described by Boyum (25). The collected blood was diluted with an equal volume (1:1) of 0.9% saline solution, slightly homogenized by inversion, and placed over 3 mL of Histopaque^®^-1077 (#10771, Sigma-Aldrich) in a Falcon tube. The tube was centrifuged at 400 *g* for 30 min at room temperature. Then, we observed the formation of a gradient with an intermediate layer composed of mononuclear cells (mainly lymphocytes and monocytes) between the plasma and Histopaque^®^-1077 layers. The cloud of cells was carefully aspirated and transferred to another tube, which was washed thrice with 0.9% saline solution and maintained in this solution until use.

### Cellular viability

Cellular viability was determined using the trypan blue dye exclusion method, as described by Strober (26).

### Protein concentration determination

Protein concentration was measured as described by Bradford (27), using the Coomassie blue method and bovine serum albumin as a standard.

### Enzymatic assay

To quantify total ADA activity, the methods described by Guisti et al. (24) and Giusti (23) were used. A standard curve was obtained using 50 mM sodium phosphate buffer (pH 7.0), ammonium sulfate, and distilled water. The incubation time and protein concentration varied from 15 to 120 min and from 1.0 to 20 μg, respectively. For pH dependence tests, 50 mM sodium acetate (pH 4.0, 5.0, and 6.0), 50 mM sodium phosphate (pH 7.0 and 7.4), or 50 mM sodium carbonate/bicarbonate (pH 8.0 and 9.0) were used. Adenosine (A9251 - Sigma-Aldrich) was used initially at 20 mM. The Michaelis-Menten constant (K_m_) and maximum velocity (V_max_) were determined from the Eadie-Hofstee plot, using the adenosine substrate at concentrations from 0.1 to 20 mM. The incubation temperature was tested at 25, 37, 45, and 60°C. For the specificity assay, adenosine, deoxyadenosine (D7400, Sigma-Aldrich), and guanosine (G6752, Sigma-Aldrich) were used. The influence of zinc (Zn^2+^), calcium (Ca^2+^), magnesium (Mg^2+^), and EDTA, at 5 mM concentrations, was determined. EHNA hydrochloride (0.1 mM) was used as an inhibitor (1261 - TOCRIS Bioscience, USA). The “blank tubes” contained 500 µL of reaction medium (20 mM adenosine/50 mM sodium phosphate buffer, pH 7.0), and the “sample tubes” contained 500 µL of reaction medium plus 25 µL of the PBMC sample suspended in saline solution. The reaction was stopped with the addition of 1500 µL of a phenol/nitroprusside solution (106 mM phenol and 0.17 mM nitroprusside) and 1500 µL of alkaline hypochlorite. After that, 25 µL of the PBMC sample was added to the “blank tubes,” and then, the tubes were incubated again for 30 min at 37°C to measure the presence of ammonium (NH_4_^+^). The enzymatic activity was determined spectrophotometrically using a Genesys 10S UV-VIS spectrophotometer (Thermo Scientific, USA) at 620 nm and reported as nmol of NH_3_·mg^-1^·s^-1^.

### Flow cytometry analysis

Three lymphocyte-rich PBMC samples were separated, washed with blocking buffer, and stained with mouse anti-human CD26-FITC conjugated antibody or an isotype control. Incubation occurred in the dark for 30 min at room temperature. After incubation, cells were washed twice with phosphate-buffered saline (pH 7.0). Then, the cells were suspended in phosphate-buffered saline (pH 7.0) and immediately analyzed using a BD Accuri C6 flow cytometer and C6 software (BD Bioscience, USA), gating on lymphocytes in the side scatter (SSC) *vs* forward scatter (FSC) dot plot.

### Statistical analysis

Data were analyzed using Student's *t*-test or one-way analysis of variance (ANOVA) followed by the Tukey's *post hoc* test. Results with P≤0.05 were considered statistically significant. The Pearson correlation was used for the linearity analysis of time and protein concentration. Kinetic parameters were calculated using GraphPad Prism 6.0 software (GraphPad Software, USA)

## Results

**Figure 1:**
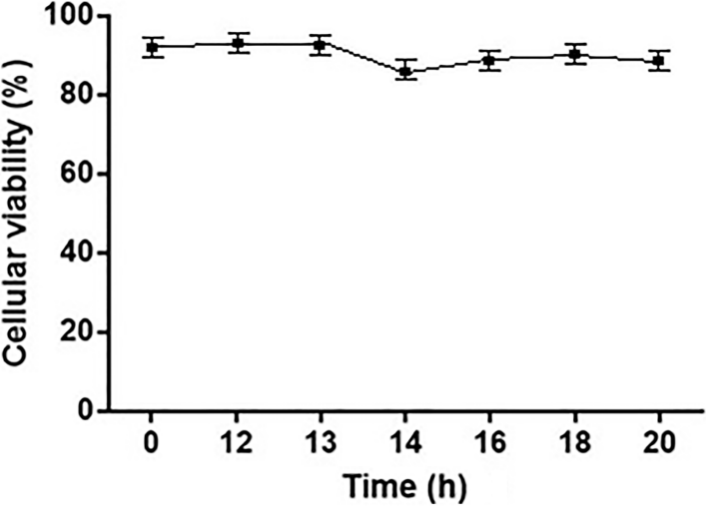
Cellular viability of human lymphocyte-rich peripheral blood mononuclear cells, determined using the Trypan Blue dye exclusion method, over a 20-h period (n=3). The data are reported as means±SE.

**Figure 2:**
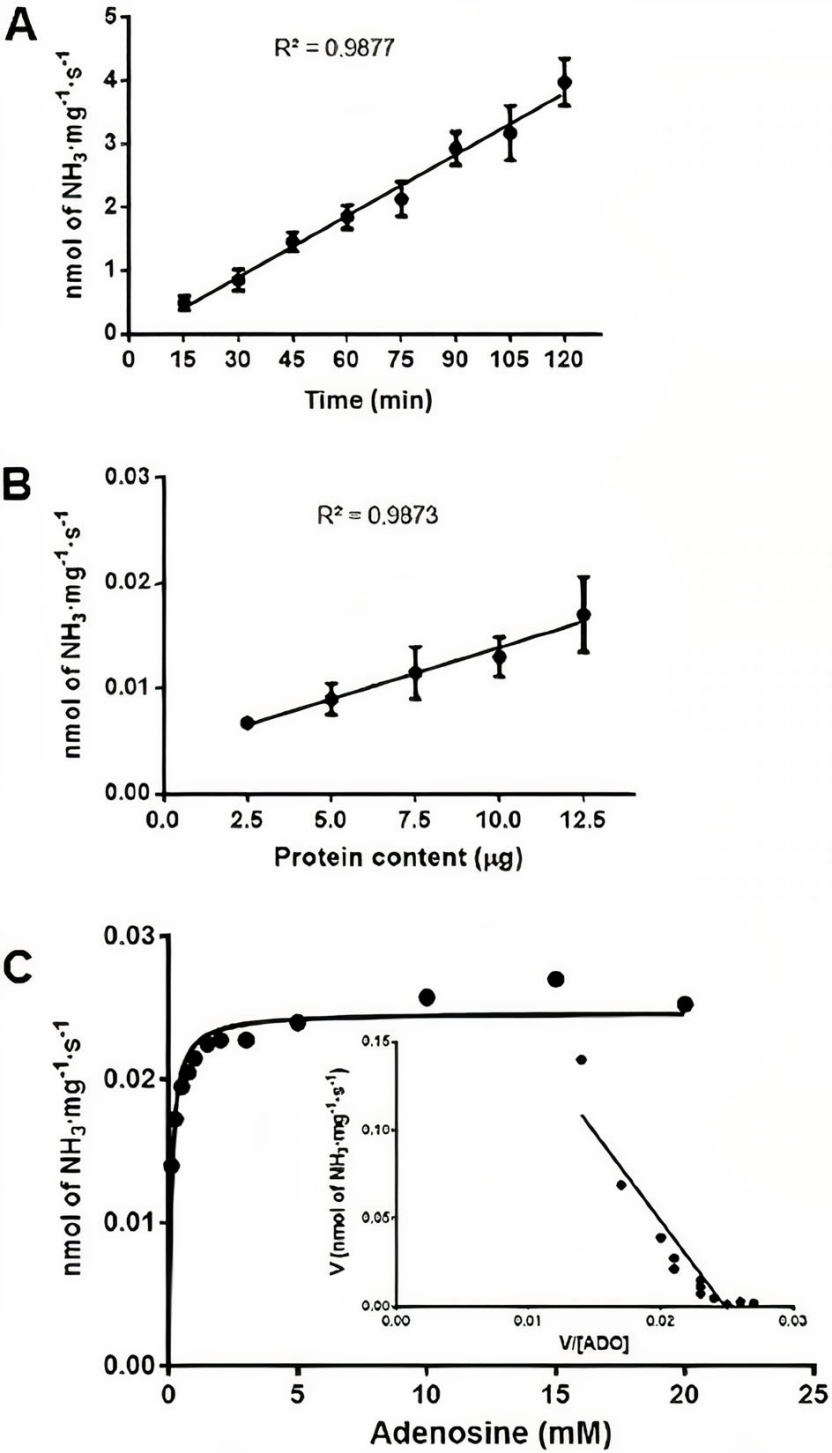
Enzymatic parameters of adenosine deaminase (ADA) activity in human lymphocyte-rich peripheral blood mononuclear cells. **A**, Effect of time on ADA activity (n=4). **B**, Effect of protein content on ADA activity (n=4). **C**, Substrate effect with Michaelis-Menten equation for determination of K_m_ and V_max_ and Eadie-Hofstee plot (n=4). The data are reported as means±SE.

The results demonstrated that human lymphocyte-rich PBMCs maintained a high viability (Figure 1), even 20 h after separation, which allowed the cells to be used in the subsequent assays, without the need for immediate enzymatic quantification after isolation. For the first ADA activity assays, 20 mM adenosine substrate was used, as recommended by Guisti et al. (24), until the ideal conditions were determined. The adenosine deamination of human lymphocyte-rich PBMCs isolated from peripheral blood was evaluated as a function of time, protein concentration, and kinetic constant, in order to determine the correct assay conditions. The enzyme activity was linear from 15 to 120 min (Figure 2A). Thus, an incubation time of 120 min was chosen for the posterior experiments. The deamination promoted by ADA activity remained linear in the range of 2.5 to 12.5 µg of protein (Figure 2B). Thus, for the following experiments, the protein content of the samples was set to values close to 12.5 µg of protein.

To calculate the kinetic parameters, we tested ADA activity at adenosine concentrations ranging from 0.1 to 20 mM. The apparent K_m_ and V_max_ were estimated with Eadie-Hofstee plots to be 0.103±0.051 mM and 0.025±0.001 nmol NH_3_·mg^-1^·s^-1^, respectively (Figure 2C). For the following tests, adenosine was used at a concentration of 10 mM, enough to achieve V_max_.

**Figure 3:**
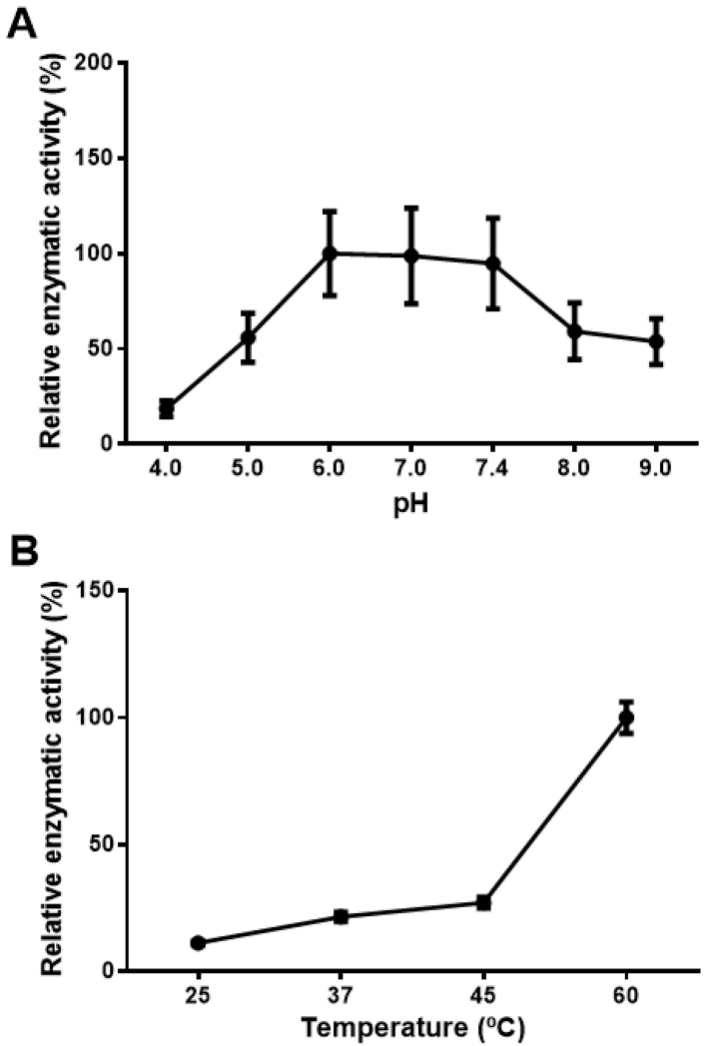
Effect of pH (**A**) and temperature (**B**) on adenosine deaminase (ADA) activity in human lymphocyte-rich peripheral blood mononuclear cells. After incubation, ADA activity was assayed under standard conditions. The data are reported as means±SE.

To determine the influence of pH on ADA activity, the enzyme assay was carried out in the pH range of 4.0 to 9.0. The relative enzymatic activity levels plateaued and remained higher in the pH range from 6.0 to 7.4 (Figure 3A). Therefore, for the following assays, sodium acetate buffer (50 mM) pH 6.0 was chosen. The effect of temperature at 25, 37, 45, and 60°C was also evaluated (Figure 3B). The relative enzymatic activity increased at 60°C; however, a temperature of 37°C was chosen in the subsequent experiments, as this is the most stable temperature for biological systems involving human cells.

**Figure 4:**
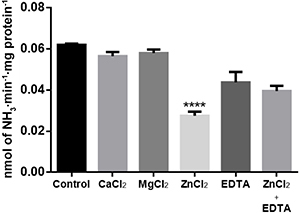
Effect of ions on adenosine deaminase (ADA) activity in human lymphocyte-rich peripheral blood mononuclear cells (n=4). The final concentration of CaCl_2_, MgCl_2_, ZnCl_2_, and EDTA was 5.0 mM. Control: without ions or EDTA. The data are reported as means±SE for n=4. ****P<0.001 compared to control group (ANOVA followed by Tukey test).

The effects of different divalent metal ions (5.0 mM CaCl_2_, MgCl_2_, or ZnCl_2_) and 5.0 mM EDTA on ADA activity were determined. ZnCl_2_ inhibited the enzyme, while the other metal ions did not affect the enzymatic activity at a concentration of 5.0 mM. EDTA (when incubated with ZnCl_2_) prevented the inhibition of the enzyme by zinc (Figure 4).

The substrate specificity of the lymphocyte-rich PBMCs ADA was evaluated (Table 1). The ADA showed the highest preference for adenosine (100%), followed by 2′-deoxyadenosine (57%) and guanosine (2.3%). Several studies have demonstrated that EHNA is a potent inhibitor of ADA1 activity (1,20). Thus, we measured adenosine deamination in human lymphocyte-rich PBMCs in the presence and absence of EHNA. The ADA activity was strongly inhibited by EHNA at a concentration of 0.1 mM (Table 2), showing only 1.7% activity after incubation with the inhibitor.

**Table 1:** Adenosine deaminase (ADA) substrate specificity in human lymphocyte-rich peripheral blood mononuclear cells (10 mM; n=4).

Substrate	ADA activity	Relative activity (%)
Adenosine	0.06354±0.00504	100±7.9
2'-deoxyadenosine	0.03636±0.00299**	57.2±4.7
Guanosine	0.00148±0.00001**	2.3±0.01

Enzymatic activity is reported in nmol of NH_3_·mg^-1^·s^-1^. The data are reported as means±SE. **P<0.01 compared to adenosine (one-way ANOVA, followed by Tukey post-test).

**Table 2:** Adenosine deaminase (ADA) inhibition in human lymphocyte-rich peripheral blood mononuclear cells by erythro-9-(2-hydroxy-3-nonyl) adenine (EHNA) (n=4).

Group	ADA activity	Relative activity (%)
Control	0.0583±0.0046	100±7.9
EHNA (0.1 mM)	0.0010±0.0001***	1.7±0.1

Enzymatic activity is reported in nmol of NH_3_·mg^-1^·s^-1^. The data are reported as means±SE. ***P<0.0001 compared to control (Student's *t*-test).

Since the association of ADA with CD26 to form a dimer has been previously described in the literature (1), PBMCs were labeled with an anti-CD26 antibody and analyzed by flow cytometry. Thus, it was possible to indirectly confirm the presence of ADA in mononuclear cells.

## Discussion

In the present study, the biochemical properties of ADA activity in human lymphocyte-rich PBMCs were evaluated. In humans, ADA is represented by two isoforms, ADA1 and ADA2, which belong to the zinc-dependent hydrolase family. The presence of these enzymes may regulate adenosine/inosine levels in human lymphocyte-rich PBMCs to modulate lymphocyte function and differentiation and inflammatory and immune responses (1,3,9).

To quantify the activity of enzymes located on the outer surface of the cell membrane, it is necessary to ensure cell integrity and viability for accurate enzymatic quantification, especially when there are intracellular enzymes that might mimic the activity of interest. ADA is located on the plasma membrane in various cells, such as lymphocytes, with its catalytic site facing the extracellular matrix (1,23). One of the most commonly used peripheral lymphocyte isolation methods is density gradient separation (25,28). The results demonstrated that human lymphocyte-rich PBMCs maintained a high viability, even 20 h after separation.

The enzyme activity was linear from 15 to 120 min, and thus, an incubation time of 120 min was chosen, and the enzyme activity remained linear in the range of 2.5 to 12.5 µg of protein. Rosemberg et al. (22) characterized ADA in the zebrafish brain and obtained linearity in enzymatic activity up to 105 min for the soluble enzyme fraction and 180 min for the membrane fraction. The same authors showed that the protein content was linear from 5.0 to 20 μg, similar to what was identified in the present study.

The substrate curve demonstrated that the apparent K_m_ and V_max_ for adenosine were 0.103±0.051 mM and 0.025±0.001 nmol of NH_3_·mg^-1^·s^-1^, respectively. The apparent K_m_ of ADA with adenosine substrate has been reported in other tissues and cells, usually presenting lower values; however, in the same order, similar to our result (17–22,29). The physiological concentration of adenosine in human plasma is approximately 0.1 µM (30), which suggests that ADA activity is very low under basal conditions (31).

The influence of pH on ADA activity was verified, and the results demonstrated that the optimal pH levels hit a plateau and remained high in the pH range from 6.0 to 7.4. Koizumi et al. (18) characterized ADA in normal human epidermal and carcinoma cells, and found that pH 7.0 was optimal. Singh and Sharma (19) characterized this enzyme in the mouse intestine, and found pH stability between 6.5 and 9.0. Additionally, Lindley and Pisoni (29) identified the optimal pH between 7.0 and 8.0 in human fibroblast lysosomes. Thus, the results obtained in this study are in agreement with the literature. The effect of temperature was also evaluated, and the relative enzymatic activity reached its maximum at 60°C. The increase in ADA activity at high temperatures has been previously described, but 37°C is commonly used in enzyme assays because of the risk of protein denaturation at higher temperatures (22). Vale et al. (21) observed higher ADA activity at 37°C and a loss of activity when the samples were incubated at 50°C. However, Koizumi et al. (18) reported that in normal human epidermal and carcinoma cells, a larger ADA isoform was stable up to 65°C.

In order to verify whether adenosine deamination in human lymphocyte-rich PBMCs is altered in the presence of divalent cations, ADA was incubated in the presence and absence of 5 mM Ca^2+^, Mg^2+^, Zn^2+^, and EDTA. The results showed that only 5 mM Zn^2+^ drastically inhibited the enzyme, and EDTA (when incubated with ZnCl_2_) prevented the effect of zinc on the enzyme. Zinc plays an important role in adenosine deamination because it is located at the active site of ADA. It has also been shown that zinc (and other divalent cations) can interact with amino acid residues and cause substrate-competitive inhibition of enzymatic activity. It has been suggested that zinc may interact with amino acid residues located at or near the active site and form a metal bond that is similar to those found in “zinc finger” structures. If so, this would occupy a portion of the active site and interfere with substrate binding (32).

In relation to the substrate specificity, ADA showed a preference for adenine over 2′-deoxyadenosin and guanine nucleosides, and the guanine nucleosides were deaminated at considerably lower rates than the adenine nucleosides (21). Iwaki-Egawa and Watanabe (20) characterized purified human and chicken liver ADAs, and their results showed that ADA has a preference for adenosine over deoxyadenosine, in agreement with the results obtained with human lymphocyte-rich PBMCs in the present study.

Since EHNA is a specific ADA1 inhibitor (1) and showed significant ADA inhibition in human lymphocytes, we can assume that ADA1 is the predominant isoform in these cells. This inhibitor has been used at the same concentration in other studies and was confirmed to have an effect on ADA1 alone, as described by Iwaki-Egawa and Watanabe (20). The human lymphocyte-rich PBMCs were labeled with anti-CD26 antibody or isotype control and analyzed by flow cytometry. Thus, it was possible to indirectly confirm the presence of ADA in PBMC.

The quantification of ADA activity in human lymphocytes has been previously performed (1,10,16), but no prior studies have performed detailed biochemical and kinetic characterizations. Thus, the present study provided the biochemical characterization of ADA in lymphocyte-rich PBMCs and identified the appropriate conditions for enzyme activity quantification. The described kinetic characteristics and conditions will improve the quantification of ADA activity in human lymphocytes in future studies and improve our understanding of the role of the purinergic system in these cells.

Finally, we highlight that the method employed in this study for cellular isolation yields a population of PBMCs rich in lymphocytes, but with the presence of non-lymphoid cells mostly composed by monocytes. Depending on the study design, this limitation should be considered.
